# Family Structure and Childhood Obesity, Early Childhood Longitudinal Study — Kindergarten Cohort

**Published:** 2010-04-15

**Authors:** Alex Y. Chen, José J. Escarce

**Affiliations:** Children's Hospital Los Angeles, Division of Research on Children, Youth, and Families; David Geffen School of Medicine at UCLA, Los Angeles, California, and RAND Health, Santa Monica, California

## Abstract

**Introduction:**

Little is known about the effect of family structure on childhood obesity among US children. This study examines the effect of number of parents and number of siblings on children's body mass index and risk of obesity.

**Methods:**

We conducted a secondary data analysis of the Early Childhood Longitudinal Study — Kindergarten Cohort (ECLS-K), which consists of a nationally representative cohort of children who entered kindergarten during 1998-1999. Our analyses included 2 cross-sectional outcomes and 1 longitudinal outcome: body mass index (BMI) calculated from measured height and weight, obesity defined as BMI in the 95th percentile or higher for age and sex, and change in BMI from kindergarten through fifth grade.

**Results:**

Other things being equal, children living with single mothers were more likely to be obese by fifth grade than were children living with 2 parents (26% vs 22%, *P* = .05). Children with siblings had lower BMI and were less likely to be obese than children without siblings. We also found that living with a single mother or no siblings was associated with larger increases in BMI from kindergarten through fifth grade.

**Conclusion:**

Children from single-mother families and, especially, children with no siblings are at higher risk for obesity than children living with 2 parents and children with siblings. These findings highlight the influential role that families play in childhood obesity. Additionally, they suggest that health care providers should consider the structure of children's families in discussions with families regarding childhood obesity.

## Introduction

For children, family represents the primary source of social learning, influence, and exposure to and adoption of health habits (1). Family provides social and interpersonal support that is instrumental in shaping and maintaining children's eating habits and physical activity patterns (1,2). Furthermore, physical and compositional characteristics of the family influence family behaviors. One key characteristic is family structure. For example, research suggests that family rules, parental involvement, sibling interactions, and emotional support — all of which are determinants of health behaviors (1-4) — are integrally linked to family structure.

More recently, studies have found that family structure also affects children's health care and physical health outcomes (5,6). Studies of childhood immunization have found that mothers who had more children were less likely to bring them for vaccination (7). Asthmatic children from single-mother families or living with 2 or more siblings had fewer visits for asthma, used fewer asthma medications, and had worse control of asthma symptoms than their counterparts in 2-parent families or living with no sibling (6). What is known about the influence of family structure on family dynamics (1,8) suggests that family structure may also affect childhood obesity.

Traditionally, the term *overweight* has been applied to children whose body mass index (BMI) is at or above the 95th percentile, on the basis of sex-specific BMI-for-age growth charts (9-13). However, expert committee recommendations (Institute of Medicine and American Academy of Pediatrics) suggest use of the term *obesity* for children with BMI percentile for age and sex in the 95th percentile or higher to reflect the correlation of high BMI with excess body fat among children and to emphasize the clinical risk of such weight status (14,15). The overall risk of becoming an obese adult is 2 to 6 times higher for children with BMI at the 95th percentile or higher (8), and childhood obesity is associated with increased risk of hypertension, hyperlipidemia, type 2 diabetes mellitus, and cardiovascular disease (16).

In this study, we used a nationally representative US dataset that tracks children from kindergarten through fifth grade to examine the effect of family structure — specifically, the number of parents and siblings in the household — on children's BMI and risk of obesity. The findings of this study have implications for health care providers and public health officials concerned about child health and childhood obesity.

## Methods

### Data source

The Early Childhood Longitudinal Study – Kindergarten Cohort (ECLS-K) is conducted by the National Center for Education Statistics using a nationally representative cohort of children who entered kindergarten during 1998-1999. The sample was drawn from approximately 1,000 schools, including public and private schools and full-day and part-day programs (17,18). Sampling was based on a multistage probability sampling design in which counties were selected, then schools, and finally children within schools. The ECLS-K collected information from children, parents, teachers, and schools by using a variety of formats, including face-to-face assessments or interviews, telephone interviews, and questionnaires (26,27). Trained assessors at the children's schools conducted the direct child assessment. Height and weight were measured in kindergarten, first grade, third grade, and fifth grade.

### Study samples

We used the ECLS-K Longitudinal Public-Use Data File, which combined data from kindergarten, first grade, third grade, and fifth grade, and included 17,565 children at baseline. We constructed the analytic sample as follows. First, we identified children in each round who were from either 2-parent or single-mother families (n = 14,831 for kindergarten, 13,123 for third grade, and 10,747 for fifth grade). Next, we included only children who had complete child direct assessment data (ie, measured height and weight) from kindergarten (n = 14,493), third grade (n = 11,855), and fifth grade (n = 10,036). Last, we included only observations with positive person-level sample weights.

### Study outcomes

The study outcomes were 1) BMI as a continuous variable, 2) obesity as a binary variable, and 3) change in BMI from kindergarten to fifth grade as a continuous variable. We used BMI to categorize children's weight status. BMI is calculated by dividing weight in kilograms by the square of height in meters (kg/m^2^) and is widely considered one of the best clinical weight criterion for children because BMI in childhood tracks well into adulthood for developing obesity (13-15). Percentile comparisons are based on the sex-specific BMI-for-age growth charts from the Centers for Disease Control and Prevention (CDC) (19,20).

### Statistical analysis

We used multivariate regression to assess the effect of family structure on the study outcomes while controlling for sociodemographic variables that may influence these outcomes. We analyzed continuous outcomes by using linear regression and binary outcomes by using logistic regression.

The main explanatory variables in the regression models were measures of family structure: an indicator variable for living in a single-mother family (vs a 2-parent family) and indicator variables for the number of siblings, categorized as none (the omitted category), 1, 2, or 3 or more. We defined 2-parent families as families in which both the father and the mother (biological, adopted, or step) were living with the child in the household. Correspondingly, we defined single-mother families as families in which the child's mother was living with the child but the father was absent. We excluded single-father families because of their small number.

The covariates in the models included the following sociodemographic characteristics: indicator variables for the child's age; sex; race/ethnicity, categorized as non-Hispanic white (the omitted category), non-Hispanic black, Hispanic, or other; family income, categorized as poor (<1.00 times the federal poverty level — omitted category), low-income (1.00-1.99 times poverty), middle-income (2.00-3.99 times poverty), or high-income (more than 4 times the federal poverty level); mother's education, categorized as high school or less (omitted), high school graduate, some college, or bachelor's degree or higher; and mother's age (at time of child's enrollment in the study), categorized as 24 years or younger (omitted), 25 to 34 years, 35 to 44 years, or 45 years or older. We used the other covariates to capture the child's health at birth: premature birth of more than 2 weeks; birth weight, categorized as less than 2,000 g (omitted), 2,000 to 2,499 g, 2,500 to 2,999 g, 3,000 to 3,499 g, and 3,500 g or more; birth order, categorized as first (omitted), second, or third or higher. We combined birth weight less than 1,500 g with birth weight less than 2,000 g because of the small sample size of very low birth weight children in the ECLS-K cohort (0.9% of the sample had birth weight <1,500 g).

We weighted all analyses by using sample weights provided by ECLS-K that reflect the sample design and survey nonresponse, and we adjusted all standard errors for clustering by using the Huber-White sandwich estimator (21,22). We conducted all analyses using Stata version 10 (StataCorp LP, College Station, Texas). This study was exempt from institutional review because it used public-use data with no identifiable information.

### Recycled prediction

To facilitate interpretation of the regression results, we used the method of recycled predictions to obtain the predicted mean values of the study outcomes for each type of family while adjusting for the covariates (6,23). Specifically, we used the estimated coefficients from the regression models to predict each outcome for each child, alternately assigning the child to each category of the family structure variable of interest (eg, single-mother vs 2-parent family) but leaving all other explanatory variables at their original values. Next, we averaged the predictions across all the children in the sample. This procedure yielded what the mean value of each outcome would be if all children in the sample lived in each particular type of family (eg, single-mother or 2-parent family) but otherwise retained the original values of all their other characteristics.

## Results

### Descriptive data

At enrollment in the study (ie, in kindergarten), excluding single-father and other family types, approximately one-fourth of the children were from single-mother families (Table 1). Nearly half of the children were girls, and most of them had at least 1 sibling. Approximately 60% of the children were non-Hispanic white, 15% were non-Hispanic black, and 18% were Hispanic.

Among all children with measured height and weight in kindergarten, mean BMI was 16.3 kg/m^2^, and 13.0% of the children were obese. In third grade, mean BMI was 18.6 kg/m^2^, and nearly 21% of the children were obese. In fifth grade, mean BMI was 20.6 kg/m^2^, and 24% of the children were obese (data not shown).

### Bivariate analyses

Family structure was significantly associated with the obesity rate (Table 2). In each grade, children from single-mother families had higher rates of obesity than children from 2-parent families. Thus, in kindergarten, 14% of children from single-mother families were obese, compared with 13% of children from 2-parent families (*P* = .05). Similarly, in third grade, 23% of children from single-mother families were obese, compared with 20% of children from 2-parent families (*P* = .03). In fifth grade, 28% of children from single-mother families were obese, compared with 22% of children from 2-parent families (*P* = .003). Moreover, in each grade the number of siblings was negatively associated with the rate of obesity. We also observed similar associations between family structure and BMI.

### Multivariate analyses

We obtained adjusted results for BMI and risk of obesity by using the multivariate models and recycled prediction (Table 3). The number of parents in the household was not associated with BMI or risk of obesity in kindergarten or third grade, but by fifth grade children from single-mother families (26%, *P* = .05) were more likely to be obese than their peers from 2-parent families (22%). In every grade we found that children with no siblings had higher BMI and a higher probability of being obese than children with siblings (Table 3).

Children living with no siblings had a larger increase in BMI than children living with siblings. The increase in BMI was 4.7 for children with no siblings; 4.2 for children with 1 sibling (*P* = .02); 3.8 for children with 2 siblings (*P* = .01); and 3.7 for children with 3 or more siblings (*P* = .006) (data not shown). However, the increase in BMI from kindergarten to fifth grade did not differ significantly by number of parents in the household.

In kindergarten, more black (14%, *P* = .02) and Hispanic (17%, *P* < .001) children were obese than were white children (12%), and more poor children were obese (15%) than were children from high-income families (10%, *P* < .001). Similarly, in third grade, more black (23%, *P* = .02) and Hispanic (26%, *P* < .001) children were obese than white children (18%), and more poor children (24%, *P* < .001) were obese than were children in high-income families (16%). Mother's education also became a significant predictor of obesity in third grade (25% for high school or less, compared with 17% for college degree or higher, *P* = .03). Results for fifth grade were similar to those for third grade.

### Sensitivity analysis

We repeated our analyses with indicator variables for the age spacing of the closest sibling, categorized as less than 1 year (omitted category), 1 to 2 years, 2 to 3 years, or 3 or more years. We found that age spacing between siblings was not associated with BMI or risk of obesity, and accounting for age spacing did not change our other findings.

### Mediation and moderation analysis

We also examined several variables, including time spent watching television (in fifth grade), fast-food consumption (in fifth grade), exercise (in fifth grade), and child care before kindergarten, to assess whether they mediated or moderated the associations of family structure with BMI and risk of obesity that we found. Among these variables, only weekly television hours (measured as the sum of hours of television, videotapes, or DVDs watched outside of school) exhibited a small mediation effect for the association of single-mother family with risk of obesity. We found no other mediation effects and no moderation effects.

## Discussion

This is the first US study to link family structure to children's BMI and childhood obesity by using a nationally representative cohort (24). Previous work has demonstrated that family structure, in addition to being a predictor of educational and developmental outcomes, also affects children's health and health care outcomes (5-7). In this study, we found strong evidence that children who lived with a single mother and especially children who had no siblings were at the greatest risk for childhood obesity. Because of the nature of our study sample, our results are likely to be generalizable to US children from 2-parent or single-mother families.

The mechanisms through which family structure affects children's weight may be related to differences across types of families in the amount of time and attention parents can devote to nurturing and providing for their children, and to the nature and extent of interactions among siblings (1-5,8,25-27). Single mothers are likely to have fewer resources, including lower availability of time and social supports, to regularly provide homemade meals for themselves and their children. Similarly, single mothers may lack the time or energy to play actively with their children and to encourage physical activities. Parents in 2-parent households may be able to spend more time with their children than single parents (28).

Whereas having more siblings has been found to have deleterious effects on other indicators of children's well-being, such as cognitive development or educational attainment, we found that having more siblings is associated with lower rates of obesity. Social scientists have observed that additional children in the family may dilute available parental time and resources (26,27). Time and resource dilution may reduce the time that parents spend reading to, teaching, or playing with each child, which could affect cognitive development (5,8,25-27) and may make it harder for parents to attend to their children's health care needs (6,25,29). Time and resource dilution may also lead parents to adopt more convenient routines, such as turning on the television and video games at home, which could result in higher rates of obesity. However, siblings may also serve as a stimulus for child-to-child interactions, cooperative play, or activities that increase the time each child devotes to physical activity. Older siblings may even serve as role models or share the caretaking role with parents. Additional siblings may decrease the availability of food for each child (thus lowering BMI), particularly for families living in poverty. Studies on the treatment of childhood obesity have demonstrated the roles that parents and family play in children's weight control and behaviors (1,2,14).

Our study has several limitations. As with any observational study, our findings may be subject to omitted variable bias from unobserved parental or family characteristics. In particular, single mothers may differ from married mothers in their attitudes about family, work, and parenting, and these differences in attitudes may influence a child's home and family environment as well as parent-child relationships. These differences in attitudes could also influence parents' choices regarding food or activity for their children. Second, we did not study single-father families or other less common family types; thus, our findings cannot be generalized to these families. Third, CDC growth charts and BMI percentiles are based on data collected when BMI-for-age was stable among children, and thus may not accurately depict current distributions of BMI-for-age (19).

Despite these limitations, our study sheds new light on the role of families in childhood obesity. Our findings suggest that children are vulnerable to increases in BMI and risk obesity in relation to the makeup of their families. A potential implication is that health care providers who treat children should plan and monitor their care in the context of family circumstances. Policies that provide additional support for single mothers, such as increased family leave time or flexible work hours, may help them to achieve healthier lifestyles for their children. Researchers and advocates should consider intervention and prevention strategies that integrate family dynamics, including the promotion of sibling interactions and collaborative activities. National and local initiatives may be more effective with the provision of additional support for single parents. School-based efforts may consider focusing on the promotion of physical activities among children without siblings. Additional research into the mechanisms underlying these relationships could be helpful in addressing childhood obesity in the United States.

## Figures and Tables

**Table 1:** Characteristics of the Sample, Early Childhood Longitudinal Study — Kindergarten Cohort, 1998-1999

**Variables**	**No. of Participants (%)^a^**
**No. of parents**	
2 parents	11,298 (76.8)
Single mother	3,195 (23.2)
**No. of siblings**	
None	2,391 (16.3)
1	6,263 (43.2)
2	3,780 (26.0)
≥3	2,059 (14.5)
**Sex**	
Girls	7,129 (48.9)
Boys	7,364 (51.1)
**Race/ethnicity^b^**	
Non-Hispanic white	8,420 (59.6)
Non-Hispanic black	1,999 (15.1)
Hispanic	2,454 (18.0)
Asian	771 (2.5)
Other	834 (4.8)
**Family income**	
Poor	2,547 (18.7)
Low-income	3,204 (23.1)
Middle-income	4,996 (33.9)
High-income	3,746 (24.3)
**Maternal education^b^**	
High school or less	1,887 (14.2)
High school graduate	4,352 (30.9)
Some college	4,680 (32.2)
College degree or more	3,564 (22.7)
**Maternal age,y^b^**	
<25	1,049 (8.0)
25-34	7,195 (50.8)
35-44	5,580 (37.1)
≥45	614 (4.1)
**Birth order**	
Firstborn	6,071 (41.2)
Second born	5,174 (36.1)
Third born or higher	3,248 (22.7)

Abbreviations: Sib, sibling; BMI, body mass index.

a Weighted by sample weights.

b Percentages do not total 100 because of missing data.

**Table 2:** Obesity Rates and Mean Body Mass Index, by Family Structure and Grade, Early Childhood Longitudinal Study, 1998-2004^a^

**Obesity Indicators, by Grade Level**	**2-Parent^b^**	**Single Mother**	*P* Value	**No Sib^b^**	**1 Sib**	***P*** Value	**2 Sibs**	***P*** Value	**≥3 Sibs**	***P*** Value
**Kindergarten**										
% Obese	12.7%	14.2%	.05^c^	14.8%	13.5%	.18^c^	11.2%	.001^c^	12.8%	.09^c^
Mean BMI, kg/m^2^	16.3	16.4	.001^d^	16.4	16.3	.02^d^	16.2	.01^d^	16.3	.03^d^
**Third Grade**										
% Obese	20.2%	22.9%	.03^c^	24.7%	21.8%	.08^c^	19.0%	.001^c^	18.9%	.003^c^
Mean BMI, kg/m^2^	18.5	18.9	.002^d^	19.1	18.7	.02^d^	18.5	0.001^d^	18.4	<.001^d^
**Fifth Grade**										
% Obese	22.4%	28.0%	.003^c^	27.8%	24.4%	.18^c^	23.1%	.07^c^	21.3%	.02^c^
Mean BMI, kg/m^2^	20.5	21.0	.006^d^	21.2	20.6	.03^d^	20.4	.005^d^	20.5	.01^d^

Abbreviations: Sib, sibling; BMI, body mass index.

a Bivariate results (unadjusted).

b Comparison category.

c Results from Pearson χ^2^ statistic corrected for the survey design.

d Results from 2-sample *t* test with sampling weights.

**Table 3:** Predicted Prevalence of Obesity and Mean BMI, by Family Structure and Grade, Adjusted for Other Covariates,^a^ Early Childhood Longitudinal Study, 1998-2004^b^

**Grade Level**	2-Parent^c^	Single Mother	*P* Value^d^	No Sib^c^	1 Sib	*P* Value^d^	2 Sibs	*P* Value^d^	≥3 Sibs	*P* Value^d^
**Kindergarten**										
% Obese	13.1%	13.0%	.91	16.2%	14.1%	.07	10.8%	<.001	11.2%	.006
Mean BMI	16.3	16.3	.41	16.5	16.4	.03	16.2	<.001	16.1	.001
**Third Grade**										
% Obese	20.4%	21.0%	.68	26.5%	22.4%	.05	18.3%	<.001	16.8%	<.001
Mean BMI	18.6	18.6	.82	19.3	18.7	.003	18.4	<.001	18.0	<.001
**Fifth Grade**										
% Obese	22.3%	26.3%	.05	27.2%	24.2%	.25	22.3%	.08	20.4%	.03
Mean BMI	20.5	20.8	.17	21.1	20.7	.12	20.2	.003	20.3	.02

Abbreviation: Sib, sibling; BMI, body mass index.

a Predicted values are adjusted for the child's age, sex, race/ethnicity, family income, mother's education and age, birth order, birth weight, and premature birth; predicted values for each family structure variable (eg, number of parents) are also adjusted for the other family structure variables.

b Multivariate results.

c Comparison category.

d Results from multivariate regression for complex survey data.
